# The Effects of Stretching Exercise on Levels of Blood Glucose: A Systematic Review with Meta-Analysis

**DOI:** 10.1186/s40798-023-00661-w

**Published:** 2024-02-09

**Authors:** Ewan Thomas, Salvatore Ficarra, Masatoshi Nakamura, Patrik Drid, Tatjana Trivic, Antonino Bianco

**Affiliations:** 1 Sport and Exercise Sciences Research Unit, Department of Psychology, Educational Science and Human Movement, University of Palermo, Via Giovanni Pascoli 6, 90144 Palermo, Italy; 2 Faculty of Rehabilitation Sciences, Nishi Kyushu University, 4490-9 Ozaki, Kanzaki, Saga 842-8585 Japan; 3 Faculty of Sport and Physical Education, University of Novi Sad, 21000 Novi Sad, Serbia

**Keywords:** Blood glucose, Diabetes, Stretching, Exercise, HbA1c

## Abstract

**Background:**

Physical activity plays an important role in the management of blood glucose levels. However, compelling evidence exists only for aerobic and resistance training. In this review, we aimed to identify the potential effects of stretching exercises on blood glucose levels.

**Methods:**

A systematic literature search was performed using the following databases: Scopus, NLM PubMed, and Web of Science. Studies regarding the effects of stretching exercise in humans on blood glucose or any related variable were included. Further inclusion criteria were: (1) original articles (published from database inception to October 2022), (2) applying stretching as a unique exercise modality, (3) having either longitudinal or acute interventions, (4) including healthy and pathological populations, and (5) having within each study a pre- and post-intervention measure. Quality assessment of the studies was conducted using the Downs and Black checklist.

**Results:**

A total of 13 articles were included. The quality assessment revealed an overall moderate quality of the included records. Ten articles included patients with type 2 diabetes (T2D), whereas the remaining three included at-risk populations. A total of 731 people with a mean age of 56.7 ± 6.1 years old were analysed. Fasting blood glucose, 2 h post-oral glucose uptake, post-stretching intervention blood glucose levels, and HbA1c were identified as variables related to blood glucose within the studies. After the stretching interventions, a significant reduction was observed in either blood glucose (ES = − 0.79; *p* = 0.0174) or HbA1c (ES = − 1.11; *p* =  < 0.0001). Meta-analytic results highlighted greater effects in T2D patients (ES = − 1.15; *p* = 0.02) and for studies applying stretching as an exercise intervention (ES = − 1.27; *p* = 0.006) rather than considering stretching as a control exercise modality.

**Conclusion:**

The results of this systematic review highlight the potential of stretching exercises to reduce blood glucose levels. In particular, if stretching is applied as a specific form of exercise intervention in patients with T2D greater effects are observed. However, further studies with more solid research designs are required, therefore, caution is needed before prescribing stretching as an exercise intervention for glycaemic management.

**Supplementary Information:**

The online version contains supplementary material available at 10.1186/s40798-023-00661-w.

## Key Points

- Stretching is an exercise modality with the potential to reduce blood glucose levels.
- Greater effects of stretching on reducing blood glucose levels are observed in Type 2 diabetic patients.
- Larger effects of stretching on reducing blood glucose were seen when stretching was the intervention investigated rather than when it was used as a comparator (control) group in studies.

**Supplementary Information:**

The online version contains supplementary material available at 10.1186/s40798-023-00661-w.

## Background

It is well established that physical activity represents an effective strategy for the prevention and management of a wide variety of diseases and conditions [1]. However, each disease, according to its pathogenesis may benefit from different forms of exercise. For example, aerobic exercise may be favoured for atherosclerosis, heart failure or hypertension [2] while resistance training (RT) may be preferred for other diseases such as multiple sclerosis or Parkinson disease [3] given that each form of exercise determines different adaptations [4]. Recently, interest in understanding the effects of exercises different from aerobic and RT for health promotion and disease management has been growing. Among these forms of exercise, the most popular are stretching, yoga, tai-chi, group activities and combined forms of exercise [5–9].

In particular, for stretching, recent evidence is shifting from the traditional focus on improving flexibility (through positioning the body or body segments in order to lengthen muscles) [10], to the broader systemic effects or evaluating specific physiological impacts of stretching interventions. For example, stretching has been observed to improve vascular stiffness, vascular endothelial function and heart rate variability concerning cardiovascular function [7, 11], to decrease nerve stiffness and pain sensitivity and increase nerve displacement concerning neural function [12], or to improve several metabolic parameters [13, 14].

An important metabolic parameter that needs to be continuously regulated is blood glucose [15]. Elevated levels of blood glucose are associated with long-term complications in a multitude of bodily systems, representing an important risk factor for several metabolic and cardiovascular diseases [16]. Optimal management of blood glucose represents a major challenge, particularly for subjects suffering from type 2 diabetes (T2D) (which is one of the world’s most common chronic diseases [17]) and metabolic syndrome [18] (another very common medically relevant condition). Among the strategies adopted to manage blood glucose, regular exercise (and particularly combined aerobic and RT interventions) represents a low-cost and very effective strategy, that can also act by providing additional beneficial effects on complications associated with T2D [19].

Despite substantial and compelling evidence regarding exercise interventions, a considerable number of patients struggle to attain the recommended levels of physical activity suggested by the American College of Sports Medicine [20]. In addition, many people suffering from chronic diseases such as T2D (and related co-morbidities) are obese, elderly or present with injuries and are not always able to autonomously perform physical activities [17]. In this regard, stretching exercises compared to other exercise modalities, can be applied in a more static context and therefore be used either in rehabilitation environments or in all those situations that would prevent individuals from autonomously exercising.

Various studies have previously examined the acute [13] or chronic effects of stretching [21] on different variables associated with blood glucose (such as fasting or post-stretching glucose levels as a measure of acute variations or glycated hemoglobin [HbA1c] as an indicator of long-term glycaemic control, reflecting the overall glycemic trends of the past two to three months [22]). However, no conclusive evidence suggests that stretching may be adopted as a form of exercise specifically for blood glucose management. Therefore, with this study, we aim to systematically review all studies investigating the effects of stretching and blood glucose (or related variables) in healthy and pathological populations. Thereafter, this review will try to identify any potential effects of stretching on blood glucose concentration or variables related to blood glucose.

## Methods

### Search Strategy

**Fig. 1:**
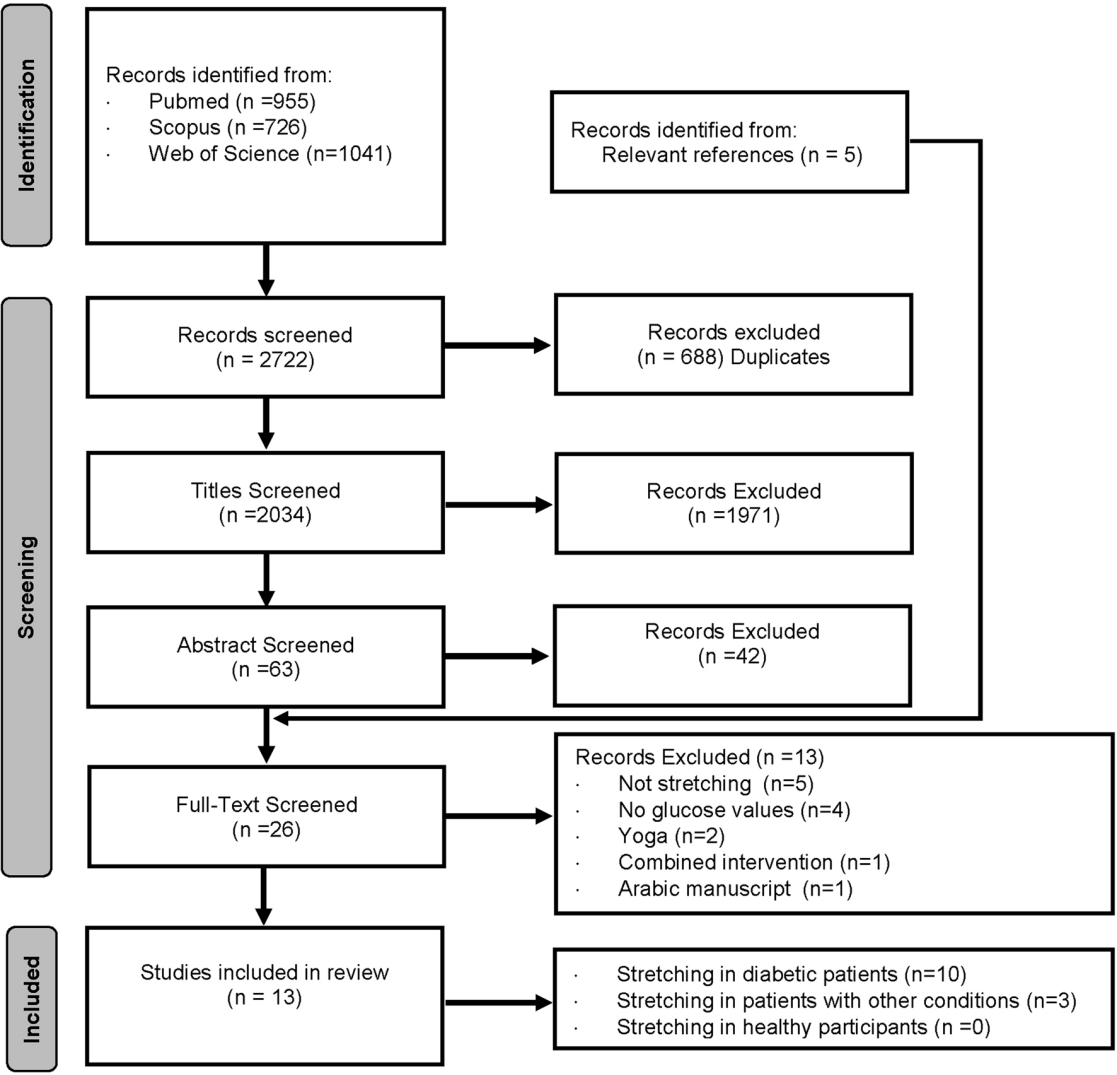
PRISMA Flow Chart illustrating the article selection process. PRISMA = preferred reporting items for systematic reviews and meta-analyses

The PRISMA guidelines for conducting a systematic review were adopted [23]. A literature search was performed using three online databases: PubMed (NLM), Scopus, and Web of Science. The search comprised articles published from database inception to October 2022. Bibliographies of relevant publications were also examined. Abstracts and unpublished materials were not included. The PRISMA flow diagram (Fig. 1) illustrates the process by which the manuscripts were selected. The full search strategy can be found in Additional file 1: Material A (Full Search Strategy).

### Inclusion and Exclusion Criteria

Studies were included for review if they fulfilled the following selection criteria: (1) original articles published in peer-reviewed journals; (2) examined the effects of stretching exercise interventions on variables pertaining to blood glucose; (3) had at least one measure of blood glucose pre-and post-intervention. All included manuscripts had to apply stretching interventions as a unique form of exercise in humans. No restrictions on the investigated population were applied (i.e., clinical, healthy, sporting, or other). Studies were excluded based on the following criteria: (1) not peer-reviewed, (2) systematic reviews and meta-analysis, (3) having only a pre-exercise or a post-exercise measure, (4) including surgical procedures, (5) combining stretching with other exercise modalities or treatments, and (6) considering yoga as a form of stretching. The PICOS model (Population, Intervention, Comparators, Outcomes, Study design) was adopted for clarity in the presentation. Details are presented in Additional file 1: Material A (Full search strategy).

Two researchers conducted the systematic search. In the event of any disagreements regarding article selection, these were ultimately resolved through a discussion with a third investigator. Abstracts and unpublished material were not included. To identify duplicated studies, manuscripts selected from each database were uploaded to the EndNote software (EndNote version X8.1; Thompson Reuters, New York, USA).

### Data Extraction

The following data were extracted from the selected studies and tabulated on a Microsoft Word file (Microsoft Corp, Redmond, USA): lead author, sample size, age of participants, stretch typology, the characteristics of the training protocols (intervention length, weekly frequency, number of sets, duration of each stretching set), the assessment methodology for blood glucose and all related variables (fasting blood glucose, blood glucose after oral consumption of sugar, post-prandial blood glucose, blood glucose post-stretching and glycated hemoglobin [HbA1c]) and the pre and post values of the blood glucose concentration. If a range of values was used, the mean was considered (e.g., 25–45 years -> 35 years). When necessary, the corresponding author of a study was contacted by the authors to request the required information. If the corresponding author did not respond with the required information before article submission, the study was not included. Table 1 reports the main characteristics of the included studies.

**Table 1:** Descriptive characteristics of the included manuscripts

Author (year)	N°	Age (years)	Type of population	Type of intervention	Blood glucose assessment	Intervention length (weeks)	Stretch typology	Sets	Time (s)	Exercises (n)	Training frequency (d/w)
Agarwal et al. [12]	40	48.9 ± 2.8	T2D	Longitudinal	NR	12	PNF	4	30	NR	3
Arsianti et al. [20]	20	> 55	T2D	Longitudinal	NR	4	P	4	30	13	3
Botton et al. [29]	26	69.7 ± 6.9	T2D	Longitudinal	Canular cubital vein sample	12	P	NR	30	NR	1
Elgayar et al. [30]	50	64.9 ± 2.8	T2D	Longitudinal	Finger prick hand-held glucometer	12	A or P	4	30	8	3
Frank et al. [33]	173	60.7 ± 6.7	PM	Longitudinal	Canular forearm vein sample	52	NR	NR	NR	NR	1
Gurudut and Rajan [31]	51	55.4 ± 7.0	T2D	Acute	Finger prick hand-held glucometer	60 min	P	NR	30	8	–
Kanaya et al. [13]	180	54.0 ± 7.0	MS	Longitudinal	Canular cubital vein sample after 2-h of a 75 g glucose load	48	P	3	30	27	3
Mehta and Patil [32]	44	40—65	T2D	Acute	Finger prick hand-held glucometer	60 min	P	3	30	4	–
Moore et al. [36]	40	70.0 ± 11.0	Stroke	Longitudinal	Canular forearm vein sample after 2-h of a 75 g glucose load	19	NR	NR	NR	NR	3
Nelson et al. [34]	22	47.0 ± 10.0	T2D or at risk	Acute	Finger prick hand-held glucometer	40 min	P	4	30	10	–
Park [35]	15	49.6 ± 5.2	T2D	Longitudinal	Canular cubital vein sample	8	P	4	30	8	3
Solomen et al. [37]	20	60.9 ± 3.1	T2D	Acute	NR	40 min	A or P	4	45	8	–
Taheri et al. [39]	50	50.7 ± 4.8	T2D	Acute	Finger prick hand-held glucometer	20 min	P	4	30	10	–
Total/mean	731	56.7 ± 6.1	–	–		14.2	–	3.8	31.4	10.7	2.5

A, Active; MS, Metabolic syndrome; NR, Not reported; P, Passive; PM, Post-menopausal; PNF, Proprioceptive neuromuscular facilitation; T2D, Type 2 Diabetes

### Quality Assessment

A modified version of the Downs and Black checklist was adopted [24]. The modified version had a maximum score of 16 (see Additional file 1: Material B, modified version of the Downs & Black checklist), where a total score of ≥ 13 (75%) indicates high methodological quality, a score of 11 or 12 (60–74%) indicates moderate quality, and a score ≤ 10 (60%) indicates low quality [25, 26]. Studies were rated independently by two authors, and the intraclass correlation coefficient was calculated to assess the measurement agreement between the two raters. The agreement between the two raters was κ = 0.91, which is considered almost perfect [27]. In the event of disagreement, a decision was reached through negotiation with the help of a third author. Individual scores for each included manuscript are reported in Additional file 1: Material C (Downs and Black Total Scores for Each Study).

### Statistical Analyses

Data from the included studies were synthesized through means and standard deviations of pre- and post-value interventions. An absolute difference for each measure was also calculated, and a percentage measure was derived for comparison across measures. Agreement between raters concerning the scoring of the quality assessment was carried out through Cohen’s kappa (κ) interrater reliability coefficient [27]. Values less than 0.59, between 0.6 and 0.79, between 0.8 and 0.9, and greater than 0.90 are indicative of weak, moderate, strong, and almost perfect reliability, respectively [27].

The following meta-analytic comparisons for the effects of stretching exercise were explored: (1) Post-Stretch versus Pre-Stretch for studies analysing glucose concentration and (2) Post-Stretch versus Pre-Stretch for studies analysing HbA1c. The following moderator analyses were explored: (1) Population (T2D vs. other included populations), and (2) Intervention objective (Stretching as intervention vs. Stretching as control). The moderator analysis could only be performed for the main analysis (1). Due to the limited number of measures a moderator analysis for stretching typology could not be performed.

Meta-analyses were performed using Jamovi (The jamovi project (2021). jamovi (Version 1.8.0.1) [Computer Software]. Retrieved from https://www.jamovi.org). For each analysis, the effect size (ES) was calculated as the difference between post-test and pre-test scores divided by the average pre-test and post-test standard deviations with Hedges’ g adjustment for small sample bias [28]. Cohen’s δ values were also calculated as the difference between post-test and pre-test scores divided by the pooled standard deviation to identify the effect size (ES) of each included intervention. The magnitude of the ES was classified according to the following scale: 0–0.19 = trivial effect, 0.20–0.49 = small effect, 0.50–0.79 = moderate effect, and ≥ 0.80 = large effect [29]. Negative effects indicated benefits (reduction in glucose or HbA1c concentration) from performing stretching. Significance was set at *p* < 0.05. Heterogeneity was explored using the I^2^ statistic, in which values < 50% indicate low heterogeneity, 50–75% moderate heterogeneity, and > 75% high heterogeneity. For studies with multiple strength analyses, the mean of the selected outcomes was used assuming dependence [28]. Data are presented as Hedges’ g ES and 95% confidence interval (95% CI). For the main analyses, results are presented considering all studies of each comparison.

## Results

The systematic search identified 2722 potential eligible articles, of which 13 were included [13, 14, 21, 30–39] (Fig. 1). The included studies comprised 731 participants with a mean age of 56.7 ± 6.1 years old. Of these, 338 were T2D patients [13, 21, 30–33, 35, 36, 38, 39], 173 were post-menopausal women [34], 180 were metabolic syndrome patients [14], and 40 were stroke patients [37]. Eight studies had a longitudinal design [14, 21, 30–34, 37], with an intervention length ranging between 4 and 52 weeks. The five remaining studies [13, 35, 36, 38, 39] had acute intervention settings and measured blood glucose from post-stretching intervention up to 60 min post-stretching intervention.

### Quality Assessment

The Downs and Black quality assessment indicated that of the 13 studies, 5 were of high quality (38.4%) [13, 14, 32, 34, 37], 2 were of moderate quality (15.4%) [33, 38] and 6 were of low quality (46.2%) [21, 30, 31, 35, 36, 39]. The mean score was 11.2 (out of 16), indicating an overall moderate quality of the included studies. The lowest scores were obtained for not knowing whether the participants of each study were representative of the entire population from which they were recruited and for not adjusting for confounding variables during the analysis. The highest scores were obtained concerning the reliability of the measured outcomes and the clear description of aims, measured outcomes, reported findings, and estimates of random variability. The results of the Downs and Black quality assessment each study can be found in Additional file 1: Material C (Downs and Black Total Scores for Each Study).

### Main Outcomes

The retrieved stretching interventions comprised either active, passive, or proprioceptive neuromuscular facilitation (PNF) stretching, applied ⁓ 2.5 times per week, through ⁓ 11 stretching exercises with stretching protocols of a mean of ⁓ 4 sets, of ⁓ 30 s duration for each set. In the majority of studies, the stretching exercises were carried out as the intervention group [13, 21, 30, 31, 33, 35, 36, 38, 39] while in others the stretching exercises were considered a control form of intervention for other exercise modalities [14, 32, 34, 37] (Table 1).

Of the included studies 6 analysed the effects of post-stretching blood glucose [13, 31, 35, 36, 38, 39] (mean reduction − 16.9%), 4 studies analysed the effects of stretching on HbA1c [14, 21, 30, 33] (mean reduction − 7.2%), 2 studies analysed the effect of stretching on fasting blood glucose [32, 34] (mean increase 2.6%), 2 studies evaluated the effects of stretching after 2-h post 75 g oral glucose uptake [14, 37] (mean reduction − 4.7%) and 1 analysed the effect of stretching on post-prandial blood glucose [33] (mean reduction − 16.4%) (Table 2). A mean reduction of 8.85% was observed for variables of blood glucose that displayed acute variations, while a mean reduction of 7.22% was observed for the only variable considering long-term variations (HbA1c).

**Table 2:** Synthesis of results for variables related to blood glucose

Author	Analyzed variable	Pre-value	Post-value	Δ	%Δ
Arsianti et al. [20]	PEBG	153.00 ± 12.47	136.10 ± 12.35	− 16.9	− 11.1
Gurudut and Rajan [31]	PEBG	250.40 ± 51.69	218.08 ± 47.08	− 32.3	− 12.9
Mehta and Patil [32]	PEBG	126.9 ± 10.37	123.40 ± 10.37	− 3.5	− 2.8
Nelson et al. [34]	40-PEBG	168.00 ± 66.00	124.00 ± 51.00	− 36	− 22.5
Solomen et al. [37]	PEBG	253.40 ± 11.03	184.60 ± 11.71	− 68.8	− 27.1
Solomen et al. [37]	PEBG	250.00 ± 24.04	166.40 ± 22.56	− 83.6	− 33.4
Taheri et al. [38]	PEBG	195.70 ± 30.10	178.90 ± 29.70	− 16.8	− 8.6
**Mean**		**199.63 ± 29.39**	**161.64 ± 26.40**	** − 36.8**	− **16.9**
Botton et al.^§^ [29]	FBG	141.61 ± 21.30	147.61 ± 31.24	+ 6	+ 4.2
Frank et al.^§^ [33]	FBG	97.40 ± 23.05	98.40 ± 22.48	+ 1	+ 1.0
**Mean**		**119.51 ± 22.18**	**123.01 ± 26.86**	**+ 3.5**	** + 2.6**
Kanaya et al.^§^ [13]	THG	7.00 ± 2.70	6.65 ± 0.68	− 0.35	− 5.0
Moore et al.^§^ [36]	THG	6.80 ± 2.60	6.50 ± 2.20	− 0.3	− 4.4
**Mean**		**6.90 ± 2.65**	**6.58 ± 1.44**	** − 0.33**	− **4.7**
Elgayar et al. [30]	PPBG	239.60 ± 27.50	192.00 ± 18.90	− 47.6	− 19.9
Elgayar et al. [30]	PPBG	235.10 ± 34.02	204.80 ± 21.50	− 30.3	− 12.9
**Mean**		**237.35 ± 30.76**	**198.4 ± 20.2**	** − 38.95**	− **16.4**
Agarwal et al. [12]	HbA1c	6.97 ± 0.19	6.77 ± 0.19	− 0.2	− 2.9
Elgayar et al. [30]	HbA1c	7.41 ± 0.60	6.65 ± 0.47	− 0.8	− 10.3
Elgayar et al. [30]	HbA1c	7.37 ± 0.60	6.78 ± 0.54	− 0.6	− 8.0
Kanaya et al.^§^ [13]	HbA1c	5.90 ± 0.40	5.50 ± 0.10	− 0.4	− 6.8
Park [35]	HbA1c	7.40 ± 1.50	6.80 ± 1.50	− 0.6	− 8.1
**Mean**		**7.01 ± 0.66**	**6.5 ± 0.56**	**− 0.5**	− **7.2**

"Bold values indicate the mean of %Δ for each analyzed variable

Δ, Difference from baseline; %Δ, Percentage difference from baseline; §, Stretching is the control intervention of the included study; 40-PEBG, 40 min post-exercise blood glucose; A, Active; BG, Blood Glucose; FBG, Fasting blood glucose; HbA1c, Glycosylated hemoglobin; P, Passive; PPBG, post-prandial blood glucose; PEBG, Post-exercise blood glucose; THG, Glucose after 2-h post 75 g oral glucose uptake

### Meta-Analysis

**Fig. 2:**
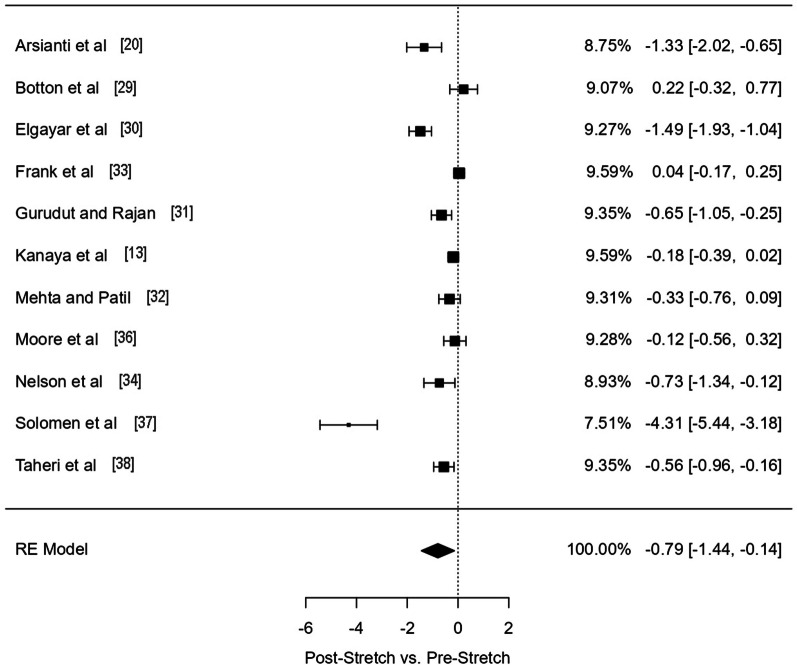
Forest Plot of studies investigating blood glucose concentration. RE: random effect. Columns in the right side of the panel represent model fitting weight% of each study, ES and [95% CI], respectively

For the comparison of Post-Stretch versus Pre-Stretch for studies analysing glucose concentration, 11 effects were included, resulting in a moderate to large significant effect on stretching in reducing blood glucose concentration (ES = − 0.79 [95% CI − 1.44, − 0.14]; *p* = 0.0174; I^2^ 96.78; *df* = 11) (Fig. 2).

When considering the analysed populations (T2D vs. other populations), a significant large effect was observed for T2D patients (ES = − 1.15 [95% CI − 2.16, − 0.13]; *p* = 0.02; I^2^ 96.56; *df* = 5) while no significant effect was observed for the other analysed populations (ES = − 0.15 [95% CI − 2.63, 0.13]; *p* = 0.18; I^2^ 49.33; *df* = 4).

Finally, subgroup analysis investigating the effects of stretching considered as an intervention form of exercise versus as a control form of exercise identified a significant effect when stretching was considered an exercise intervention (ES = − 1.27 [95% CI − 2.19, − 0.36]; *p* = 0.006; I^2^ 95.6; *df* = 7) while no significant effect was observed when stretching was considered a control intervention (ES = − 0.05 [95% CI − 0.22, 0.11]; *p* = 0.53; I^2^ 22.6; *df* = 4).

**Fig. 3:**
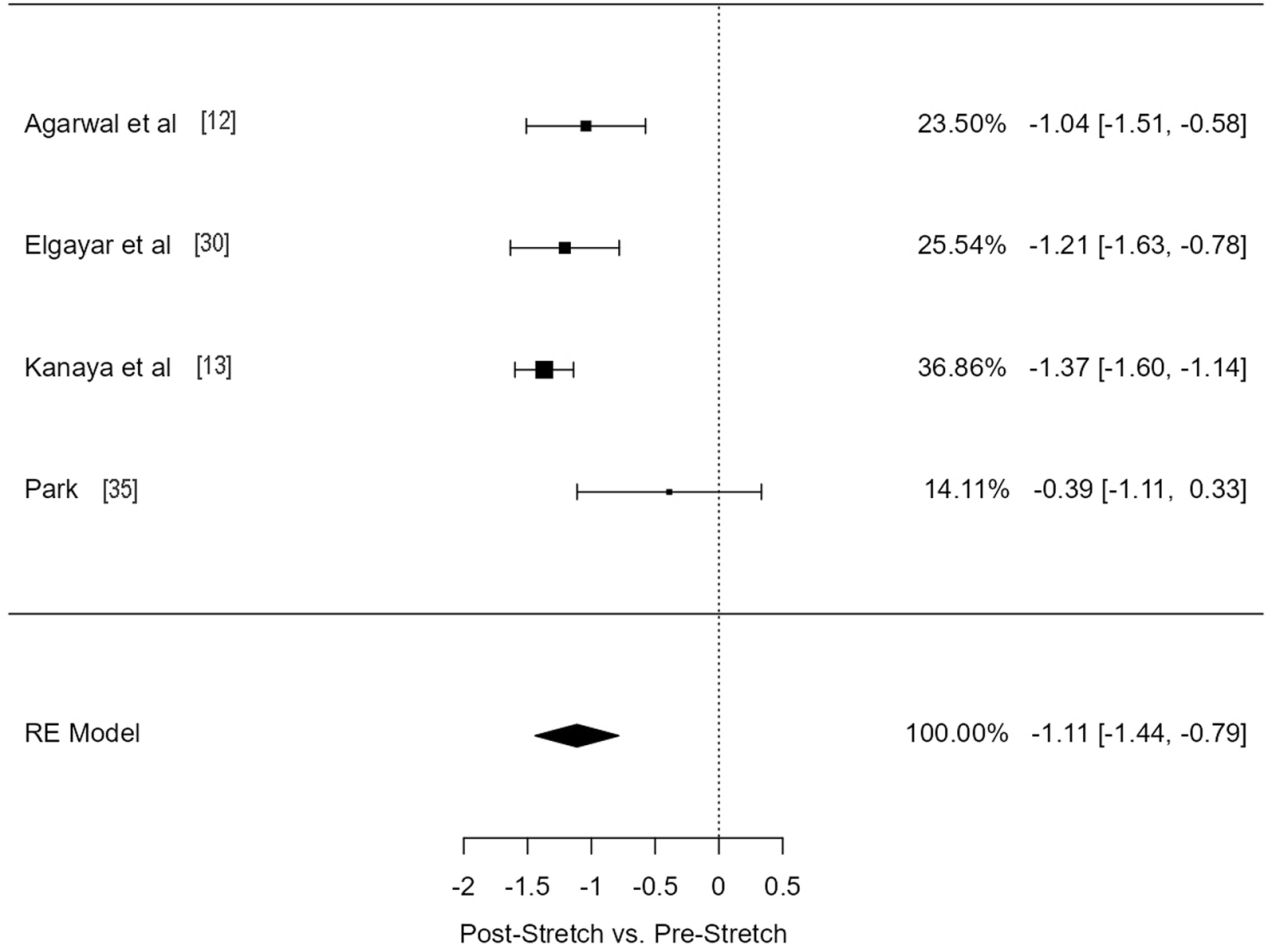
Forest Plot of studies investigating levels of HbA1c. RE: random effect. Columns in the right side of the panel represent model fitting weight% of each study, ES and [95% CI], respectively

For the comparison of Post-Stretch versus Pre-Stretch for studies analysing HbA1c, 4 effects were included, resulting in a significant large effect on stretching in reducing HbA1c (ES = − 1.11 [95% CI − 1.44, − 0.79]; *p* =  < 0.0001; I^2^ 58.26; *df* = 4) (Fig. 3).

## Discussion

This systematic review, accompanied by a meta-analysis aimed to understand the effects of stretching exercises on blood glucose concentration. Our findings revealed that stretching has the potential to reduce blood glucose concentration, whether measured post-exercise, after a period of fasting, after 2 h of a 75 g glucose intake, or when considering the levels of HbA1c. Notably, our focus shifts to the results of the moderation analysis, which highlights that greater effects were seen in patients with T2D compared to other screened populations (metabolic syndrome patients, post-menopausal women, stroke patients, or patients at risk of T2D). Furthermore, all interventions that employed stretching as the primary form of exercise intervention, irrespective of study duration, showed significant effects in reducing blood glucose concentration. Conversely, studies using stretching as a control form of exercise intervention showed no such effects. Despite the interesting results, caution is warranted, since many of the included studies are deemed of low quality. Thus, further high-quality studies with solid research designs are needed before performing stretching exercises, especially if such an exercise modality is chosen as a stand-alone form of intervention, in pathological or at-risk populations.

Existing evidence on physical activity, generally concurs that exercise (either aerobic or RT) is able to acutely and chronically reduce blood glucose [40]. The results of this systematic review align with those of other exercise modalities. Noteworthy is a study included in this systematic review, that compared the effects of stretching to those of a RT intervention [35] in terms of acute effects on blood glucose post-exercise. The study’s findings highlight that both interventions were able to reduce blood glucose, with no significant difference between the applied protocols (Stretching vs. RT, − 12.9% vs. − 20.1, respectively).

The majority of the included studies evaluated post-stretching blood glucose [13, 31, 35, 36, 38, 39]. Among these, only one adopted a longitudinal intervention lasting 4-weeks [31] while the remaining employed acute interventions ranging from 20 to 60 min in duration [13, 35, 36, 38, 39]. All studies investigating post-stretching blood glucose focused on T2D patients or those at risk of T2D [38]. When analysing the mean values across interventions, the measured blood glucose (assessed using a handheld glucometer from a finger-prick blood drop in all studies) averaged 199.44 mg/dl. Across all interventions, a decrease was observed, resulting in a mean post-intervention value of 161.64 mg/dl (− 16.9%) (It should be noted that both pre and post-values fall within physiological ranges for exercise recommendations [41]). However, there were substantial differences in the blood collection protocols. In four of the included studies [31, 35, 36, 39], participants were instructed to eat a meal, therefore the post-stretching blood glucose was also a post-prandial measure (pre 206.5 mg/dl vs. post mean value 165.72 mg/dl). In the two remaining studies [13, 38] this information was not provided (pre 181.8 mg/dl vs. post mean value 151.5 mg/dl).

Only two of the studies included in the analysis assessed fasting blood glucose [32, 34]. Both of these carried out longitudinal interventions ranging between 12 and 52 weeks. However, in both studies, the stretching intervention was not considered the primary form of exercise but rather a control intervention. Unfortunately, these studies offer minimal information regarding the stretching protocol or the adopted exercises. However, a common pattern in both studies is that the stretching intervention was applied once weekly, which according to the recommendations provided by a previous systematic review on optimal stretching dose–response [42] appears to be insufficient for long-term interventions. In both studies, blood glucose was measured through the collection of blood from venous access to the cubital region of the arm. In Botton’s et al. study [32] (T2D diabetic participants) the mean pre-value was 141.61 mg/dl, while the mean post-value was 147.61 mg/dl. In Frank’s et al. study [34] (post-menopausal women) the mean pre-value was 97.4 mg/dl while the mean post-value was 98.4 mg/dl. In both studies, no decrease in glucose levels was observed (conversely, a slight increase is present 2.6%). This observation suggests that despite the provision of a stretching intervention to participants, factors such as frequency and exercise parameters (such as the number of exercises, sets, and stretching duration (which were not reported)) might not have been sufficient to elicit an observable response in the measured parameters. Regarding the effects of stretching after 2 h post 75 g oral glucose uptake, only two studies were identified [14, 37]. Similarly to the previous section, the two studies included stretching exercises as a control intervention and limited information regarding the stretching protocols are reported. Both studies had longitudinal designs ranging between 19 and 48 weeks. Blood samples in both studies were collected by venous access to the arm cubital area after instructing the participants to fast overnight (12 h). The collection was then performed two hours after a standard 75 g oral glucose load. The mean pre-value reported for both studies was 6.9 mmol/l (124.2 mg/dl, which is considered a normal reference value for glucose tolerance [43]) with a post-value of 6.58 mmol/l (118.35 mg/dl). In both studies, a small and not clinically meaningful mean decrease has been reported. It is interesting to note that the other included interventions (yoga and the combined aerobic/anaerobic exercise program) show similar outcomes, with a mean pre-value of 7.2 mmol/l (129.6 mg/dl) and a mean post-value of 6.73 mmol/l (121.4 mg/dl). Both studies were not conducted on T2D patients. The only study evaluating post-prandial blood glucose [33], directly compared active to passive stretching in diabetic patients. The measurement of blood glucose was performed post-prandially before and at the end of the 12-week intervention with an Accu-Chek hand held glucometer. The mean pre-value was 237.35 mg/dl which decreased to 198 mg/dl after the stretching interventions. It is interesting to note that despite the authors' report that no significant differences between the two stretching modes were present, a relatively greater effect was observed after active stretching (19.9 vs. 12.9% decrease, compared to passive stretching, respectively). The results of the study by Elgayar et al. reveal that post-intervention values decreased from “diabetic” (above 200 mg/dl [41]) to pre-diabetic reference ranges (140 and 199 mg/dL for post-prandial values [41]). Recent evidence has evaluated the effects of different exercise modalities (aerobic, RT and a combination of both) on glucose response in diabetic patients [44, 45]. Evidence revealed that all the exercise types improved the post-meal glycaemic response with no differences between aerobic and RT. Notably, it is intriguing that the percentage reductions observed after other exercise modes fall within 6 to 30% for post-prandial values, which are (as for other glucose measurements) almost overlappable with those above reported from the stretching interventions (12.9 and 19.9%).

In a previous systematic review, we aimed to understand the effects of stretching on the cardiovascular system [7] and identified positive effects on vascular endothelial function. The vascular endothelium is fundamental for glucose transport from the bloodstream to peripheral tissues [46] through a dynamically regulated vasodilation process. Flow-mediated dilation [47], is a commonly employed measure for assessing endothelial function, where compromised vascular dilation is indicative of endothelial dysfunction. It is also well known that a continuous hyperglycemic state, frequent glycaemic peaks and the presence of diabetes are associated with endothelial dysfunction [48, 49]. Furthermore, it is worth noting that endothelial dysfunction has been observed to manifest even prior to the onset of a fully developed hyperglycemic state, both in healthy individuals and in patients with T2D [47]. Therefore, for patients with T2D or insulin resistance, targeting endothelial dysfunction early on could emerge as a pivotal strategy for preventing cardiovascular damage linked to prolonged hyperglycemia [48]. Common strategies employed to improve endothelial dysfunction are pharmacological, nutritional, hormonal and exercise therapies [50]. In this context, the reduction in blood glucose concentration observed post-stretching, as evidenced in this systematic review, gains additional significance, particularly within the T2D studies (ES = -1.15; *p* = 0.02). This reduction may also be a consequence of the improvement of vascular endothelial function [7] caused by the stretching interventions. Stretching stimuli have been seen to act on vascular endothelium by increasing Ca^+^ release, which would then act by increasing the release of nitric oxide (NO) [51] which has been observed (in cultured cells) to increase by around 20% after a single passive stretch [52]. Previous studies have shown that both Ca^+^ and NO are involved in signal transduction mechanisms that act by increasing glucose transport after exercise [53, 54]. Another observed effect induced by stretching in vivo is a blood flow hemodynamic change, with decreased blood flow (causing transient ischemia) during the stretch phase and enhanced blood flow (hyperemia) immediately after the stretch [51]. This phenomenon potentially introduces another mechanism influencing blood glucose concentration. Although ischemia has been shown to act by decreasing glucose uptake [55], conversely, post-exercise hyperemia determines increased glucose uptake in skeletal muscle cells [56]. A further possible mechanism responsible for glucose reduction post-stretching would involve an increase in its primary transporter, GLUT-4 [57]. The GLUT-4 transporter is very sensitive to external stimuli, in particular, the presence of insulin and exercise increase the number of transporters within skeletal muscles [58]. A decline in high-energy phosphates (caused by exertion), and an increase in Ca^+^ are other mechanisms mediated by exercise that act by increasing the transcription of the glucose transporter [58]. These mechanisms which all together indicate increased energy expenditure are able to increase the GLUT-4 transporter in muscle cells. Therefore, stretching may up-regulate such transporters by increasing energy expenditure similarly to other exercise modalities [59]. Although no evidence exists in humans concerning the effects of stretching on GLUT-4 expression, animal studies have shown that stretching can increase the transcription of the glucose transporter [60].

All the above-mentioned results refer to blood glucose concentrations which regardless of the duration of the interventions, offer an assessment of glycemic status of individuals at the specific measurement moment [22]. For this reason, a distinction between blood glucose values and HbA1c had to be performed.

Regarding the results of HbA1c, of the four included studies [14, 21, 30, 33], three were performed on diabetic patients (mean value 7.29%) [21, 30, 33] and one on patients with metabolic syndrome (mean value 5.9%) [14]. The mean pre-intervention value across the studies was 7.01% (which according to the international reference values may be considered a diagnostic criterion for diabetes when HbA1c is > 6.5% [41]). Results of the meta-analysis revealed that a significant and large reduction in HbA1c was observed (ES = − 1.11; *p* =  < 0.0001). The applied stretching included either active or passive static stretching and PNF which were applied in protocols ranging from 8 to 48 weeks. The mean post-intervention stretching value for HbA1c was 6.5% (6.75% for diabetic patients and 5.5% for patients suffering from metabolic syndrome). Although the results highlight a reduction of HbA1c in all the included studies (mean percentage decrease of 7.22), it is notable that the value only falls within the physiological range for patients with metabolic syndrome. However, it is important to mention that this value lies near the upper limit of this range. It is crucial to emphasize that HbA1c reflects long-term glycaemic history, therefore the length of the intervention could be an important factor in order to appraise changes from the applied interventions (two interventions were carried out over a 12-week period [30, 33], one over 8 weeks [21] for the T2D population and only the intervention carried out in metabolic syndrome patients [14] was over a 48 week period).

In other studies investigating the effects of exercise on HbA1c in diabetic patients, aerobic and anaerobic activities have been evaluated. In particular, after both aerobic [61] and RT [62] or studies comparing aerobic to RT [63, 64] the values of HbA1c decrease. These results were not observed in the associated control groups. A further aspect to be considered is that when different exercise modes are combined, greater effects are generally observed [19]. To be noted, to date no evidence is available regarding interventions for glycaemic control in which stretching exercises and other exercise modes are combined. However, for both aerobic and RT a return to physiological values after the intervention periods was not observed in T2D patients [61–64].

Notwithstanding that this review with meta-analysis has highlighted a reduction in parameters related to blood glucose concentration and HbA1c, information regarding the effects of stretching on insulin sensitivity is lacking. Only three of the included studies [14, 34, 37] reported results regarding insulin sensitivity indexes in which, however, no variation was observed (HOMA index pre-4.3 vs. post 4.4). It should be noted that all three studies used stretching as a control form of exercise.

The main limitations of this study are the relatively small number of included studies (n = 13) and the overall quality of the included records, with many of these being of low quality. In addition, since not every study had a control group, the meta-analysis was performed between pre and post-measures, and therefore, studies with more solid designs (i.e. randomised controlled trials) are needed to verify the results obtained by the present investigation. Further, the included studies provided an overview of the effects of the stretching interventions on blood glucose concentration, however, almost no information concerning physiological mechanisms or the effects of stretching on insulin sensitivity is reported. We believe that the heterogeneity observed among the interventions (i.e. static, active or PNF stretching in acute and longitudinal protocols) may be a strength of this review since the results of the included records, regardless of the intervention characteristics, show that stretching has the potential to reduce blood glucose concentration. It is also important to note that although our objective was to discern the effects of stretching exercises on blood glucose in both healthy and pathological populations, no studies were identified that included healthy subjects. Future research should encompass individuals without underlying health conditions to provide a more comprehensive understanding of the impact of stretching exercises on blood glucose.

The effects of stretching in diabetic and at-risk populations seem to be similar to those of other well-investigated exercise modalities (aerobic and RT). However, stretching holds the potential advantage of being applicable passively to individuals who are unable to engage in active exercise, providing an alternative for those who cannot engage in more dynamic exercises for managing glycemic control.

## Conclusion

The results of this systematic review highlight that stretching interventions, despite their heterogeneity in terms of stretching types, have the potential to decrease blood glucose concentration and HbA1c levels. Nevertheless, these effects seem to be present only in type 2 diabetic patients when compared to other pathological or at risk conditions.

One distinctive advantage of stretching in comparison to other exercise modes is its capacity to be passively applied. However, further studies with more solid research designs are required; therefore, caution is needed before prescribing stretching as an exercise intervention for glycaemic management.

### Supplementary Information

**Additional file 1**. **Material A.** Full Search Strategy. **Material B.** The modified version of the Downs & Black checklist. **Material C.** Downs and Black Total Scores for Each Study.

## Data Availability

Full data coded of the included studies can be shared upon reasonable request from the corresponding author.

## Abbreviations

95% CI: 95% Confidence interval
ES: Effect size
HbA1c: Glycated haemoglobin
NO: Nitric oxide
PICOS: Population, intervention, comparison, outcome and study type
PNF: Proprioceptive neuromuscular facilitation
PRISMA: Preferred reporting items for systematic review and meta-analysis
RT: Resistance training
T2D: Type 2 diabetes
