# Supplementary material for: The Effects of Stretching Exercise on Levels of Blood Glucose: A Systematic Review with Meta-Analysis
Source: Sports Med Open. 2024 Feb 9;10:15. doi: 10.1186/s40798-023-00661-w (PMC10858005; doi:10.1186/s40798-023-00661-w)
Supplement: Supplementary file 1 — Additional file 1. Material A. Full Search Strategy. Material B. The modified version of the Downs & Black checklist. Material C. Downs and Black Total Scores for Each Study. [file 40798_2023_661_MOESM1_ESM.docx]

**Material A.** Full Search Strategy.

**Keywords**

1. Stretching

2. Blood glucose

3. Blood sugar

4. Glycemia

5. Glycated haemoglobin

6. Insulin Resistance

7. Diabet*

| **PICOS** |
| --- |
| **Population** |
| Healthy and pathological individuals |
| **Interventions** |
| Stretching exercises (Static stretching, either active or passive and Proprioceptive Neuromuscular Facilitation) |
| **Comparators** |
| Post-exercise blood glucose levels were compared to pre-exercise blood glucose levels within each study |
| **Outcomes** |
| Blood glucose levels (Fasting, post-2-h 75g oral glucose uptake, post-prandial, HbA1C) |
| **Study design** |
| Original articles (Acute and longitudinal interventions) |
| **Limitations** |
| Only peer-review articles published up to October 2022, no systematic reviews and meta-analysis, no articles performing surgery, no articles with only a pre-exercise or post exercise measure, no articles combining stretching to other exercise modalities (Aerobic, RT, Tai-chi, group activities, yoga) or other treatments (body mind interventions, rehabilitation), and no studies considering yoga as a form of stretching. |

Keywords were arranged into two subsets. Subset A comprised the keyword: stretching. Subset B comprised the keywords: Blood glucose, Blood sugar, Glycemia, Glycated haemoglobin, Insulin Resistance, Diabet*. Group A keywords were used and combined through Boolean operators with all group B keywords.

Search array: (stretching AND blood glucose) OR (stretching AND diabet*) OR (stretching AND glycated haemoglobin) OR (stretching AND glycemia) OR (stretching AND blood sugar) OR (stretching AND insulin resistance)

The search was conducted from database inception up to the 10th of October 2022.

**Material B.** The modified version of the Downs & Black checklist.

| **Item** | **Yes** | **Unable to determine** | **No** |
| --- | --- | --- | --- |
| 1. Is the hypothesis/aim/objective of the study clearly described? | 1 |  | 0 |
| 2. Are the main outcomes to be measured clearly described in the Introduction or Methods section? | 1 |  | 0 |
| 3. Are the characteristics of the patients included in the study clearly described? | 1 |  | 0 |
| 5. Are the distributions of principal confounders in each group of subjects to be compared clearly described? | 2 | Partially 1 | 0 |
| 6. Are the main findings of the study clearly described? | 1 |  | 0 |
| 7. Does the study provide estimates of the random variability in the data for the main outcomes? | 1 |  | 0 |
| 10. Have actual probability values been reported (e.g. 0.035 rather than <0.05) for the main outcomes except where the probability value is less than 0.001? | 1 |  | 0 |
| *External Validity* |  |  |  |
| 11. Were the subjects asked to participate in the study representative of the entire population from which they were recruited? | 1 | 0 | 0 |
| 12. Were those subjects who were prepared to participate representative of the entire population from which they were recruited? | 1 | 0 | 0 |
| *Internal Validity - Bias* |  |  |  |
| 16. If any of the results of the study were based on “data dredging”, was this made clear? | 1 | 0 | 0 |
| 18. Were the statistical tests used to assess the main outcomes appropriate? | 1 | 0 | 0 |
| 20. Were the main outcome measures used accurate (valid and reliable)? | 1 | 0 | 0 |
| *Internal Validity – Confounding (selection bias)* |  |  |  |
| 21. Were the subjects in different intervention groups (trials and cohort studies) or were the cases and controls (case-control studies) recruited from the same population? | 1 | 0 | 0 |
| 22. Were study subjects in different intervention groups (trials and cohort studies) or were the cases and controls (case-control studies) recruited over the same period of time? | 1 | 0 | 0 |
| 25. Was there adequate adjustment for confounding in the analyses from which the main findings were drawn? | 1 | 0 | 0 |

**Material C.** Downs and Black Total Scores for Each Study.

| **Authors** | **1** | **2** | **3** | **5** | **6** | **7** | **10** | **11** | **12** | **16** | **18** | **20** | **21** | **22** | **25** | **Total** | **Q** |
| --- | --- | --- | --- | --- | --- | --- | --- | --- | --- | --- | --- | --- | --- | --- | --- | --- | --- |
| Agarwal et al | 1 | 1 | 1 | 0 | 1 | 1 | 1 | 1 | 0 | 1 | 0 | 1 | 1 | 0 | 0 | 10 | L |
| Arsianti et al | 1 | 1 | 0 | 0 | 1 | 1 | 1 | 0 | 0 | 1 | 0 | 1 | 0 | 0 | 0 | 7 | L |
| Botton et al | 1 | 1 | 1 | 1 | 1 | 1 | 1 | 1 | 0 | 1 | 1 | 1 | 1 | 1 | 1 | 14 | H |
| Elgayar et al | 1 | 1 | 1 | 0 | 1 | 1 | 1 | 0 | 0 | 1 | 1 | 1 | 1 | 1 | 0 | 11 | M |
| Frank et al | 1 | 1 | 1 | 2 | 1 | 1 | 1 | 1 | 0 | 1 | 1 | 1 | 0 | 1 | 1 | 14 | H |
| Gurudut and Rajan | 1 | 1 | 1 | 1 | 1 | 1 | 1 | 1 | 0 | 1 | 0 | 1 | 0 | 0 | 0 | 10 | L |
| Kanaya et al | 1 | 1 | 1 | 2 | 1 | 1 | 1 | 1 | 0 | 1 | 1 | 1 | 0 | 1 | 1 | 14 | H |
| Mehta and Patil | 1 | 1 | 0 | 0 | 1 | 1 | 1 | 0 | 0 | 1 | 1 | 1 | 1 | 0 | 0 | 9 | L |
| Moore et al | 1 | 1 | 1 | 2 | 1 | 1 | 1 | 1 | 0 | 1 | 1 | 1 | 0 | 1 | 0 | 13 | H |
| Nelson et al | 1 | 1 | 1 | 1 | 1 | 1 | 1 | 1 | 0 | 1 | 1 | 1 | 0 | 0 | 0 | 11 | M |
| Park | 1 | 1 | 1 | 0 | 1 | 1 | 0 | 0 | 0 | 1 | 1 | 1 | 1 | 0 | 0 | 9 | L |
| Solomen et al | 1 | 1 | 1 | 0 | 1 | 1 | 1 | 0 | 0 | 1 | 1 | 1 | 1 | 0 | 0 | 10 | L |
| Taheri et al | 1 | 1 | 1 | 1 | 1 | 1 | 1 | 1 | 0 | 1 | 1 | 1 | 1 | 1 | 1 | 14 | H |
| **Overall score** |  |  |  |  |  |  |  |  |  |  |  |  |  |  |  | **11.2** | **M** |
| Overall quality L: Low, ≤10; M: Moderate, 11 or 12; H: High, ≥13. | | | | | | | | | | | | | | | | | |
